# Factors associated with condomless anal sex among adolescent men who have sex with men and transgender women in three Brazilian state capitals: a PrEP1519 study

**DOI:** 10.11606/s1518-8787.2024058005462

**Published:** 2024-10-11

**Authors:** Rijone Rosário, Inês Dourado, Marcos Pereira, Lorenza Dezanet, Dirceu Greco, Alexandre Grangeiro, Laio Magno

**Affiliations:** I Universidade Federal da Bahia. Instituto de Saúde Coletiva. Salvador, BA, Brasil Universidade Federal da Bahia Instituto de Saúde Coletiva Salvador BA Brasil; II Fundação Oswaldo Cruz. Instituto René Rachou. Belo Horizonte, MG, Brasil Fundação Oswaldo Cruz Instituto René Rachou Belo Horizonte MG Brasil; III Universidade Federal de Minas Gerais. Faculdade de Medicina. Belo Horizonte, MG, Brasil Universidade Federal de Minas Gerais Faculdade de Medicina Belo Horizonte MG Brasil; IV Universidade de São Paulo. Faculdade de Medicina. São Paulo, SP, Brasil Universidade de São Paulo Faculdade de Medicina São Paulo SP Brasil; V Universidade do Estado da Bahia. Departamento de Ciências da Vida. Salvador, BA, Brasil Universidade do Estado da Bahia Departamento de Ciências da Vida Salvador BA Brasil

**Keywords:** Men who have Sex with Men, Transgender Women, Adolescent, Condomless anal sex, HIV/AIDS, Sexually Transmitted Infections

## Abstract

**OBJECTIVE:**

To analyze the factors that increase the practice of condomless anal sex (CAS) among adolescent men who have sex with men (AMSM) and adolescent *travestis* and transgender women (ATGW) in three Brazilian state capitals.

**METHODS:**

PrEP1519 is a prospective, multicenter cohort study demonstrating the effectiveness of human immunodeficiency virus (HIV) pre-exposure prophylaxis (PrEP) among AMSM and ATGW aged from 15 to 19 years in three Brazilian state capitals. The analyses were performed with baseline cohort data, including 1,418 adolescents enrolled from 2019 to 2021. The outcome studied was CAS in the last six months, and the potentially associated factors were sociodemographic, behavioral, healthcare, and history of violence and discrimination. Descriptive, bivariate, and multivariate analyses were conducted. Adjusted prevalence ratios (aPRs) and 95% confidence intervals (95%CI) were estimated.

**RESULTS:**

Most of the participants were AMSM (91.5%), aged 18 to 19 years (75.9%), Black (40.5%), with secondary or higher education in progress (92.7%), with CAS during the first sexual intercourse (54.2%), sexual initiation before the age of 14 (43.4%), and history of group sex (24.6%) and transactional sex (14.6%). The prevalence of CAS in the last six months was 80.6% (95%CI 78.5%-82.6%). Adolescents who reported condomless first sexual intercourse (aPR: 1.18; 95%CI 1.10-1.25), use of psychoactive substances (aPR: 1.09; 95%CI 1.03-1.16), and transactional sex (aPR: 1.11; 95%CI 1.04-1.20) had a higher prevalence of CAS in the last six months. We also found that those aged 15 to 17 years had a higher prevalence of CAS than those aged 18 to 19 (aPR: 1.07; 95%CI 0.99-1.13).

**CONCLUSIONS:**

The prevalence of CAS was high among AMSM and ATGW, being associated with practices that may increase the risk of sexually transmitted infections (STIs). Therefore, it is recommended to strengthen sexual health programs for young people that address the issue of sexuality and STI prevention, as well as to expand access to preventive methods, such as condoms and PrEP.

## INTRODUCTION

Adolescence is a phase of life characterized by physical, psychological, and cognitive changes and the expansion of social interactions. In this period, adolescents are faced with demands, challenges, and new experiences^1^. In this sense, sexual health education is recommended to promote information that improves quality of life^2^.

However, sexual health education for adolescents and youth remains a challenge for families and education and health systems, especially in low- and middle-income countries^3^. Brazil, for example, has registered several obstacles in the conduct of sex education programs in schools in recent years, primarily due to social conservatism^4^. A study conducted in Salvador, Bahia, from 2017 to 2018 highlighted weaknesses in the process of developing and integrating sexual health education activities in public schools, mainly by cause of the difficulty of dialogue between the health (i.e., Brazilian Family Health Strategy) and education (i.e., public schools) sectors within the scope of the Brazilian School Health Program (*in Portuguese: Programa Saúde na Escola* - PSE). Moreover, that study recorded experiences of racism and discrimination related to homosexuality experienced by adolescents in the school environment^3^.

In this context, Brazil has registered an increase in the incidence rate of human immunodeficiency virus (HIV) among adolescents and young people^5^. The Brazilian Ministry of Health also shows particular concern about men who have sex with men (MSM) and *travestis* and transgender women (TGW) as epidemiological surveillance studies have estimated high prevalence of HIV in these populations. For instance, two national surveys conducted among MSM recorded an increase in HIV prevalence, from 14.2% in 2009 to 18.4% in 2016^6^^), (^^7^. Among TGW, HIV prevalence is disproportionately higher than that recorded among the general population (0.6%) from 2001 to 2021^8^, with substantial regional and temporal differences: 9% from 2014 to 2016 and 24.3% from 2016 to 2017 in Salvador^9^ and 31.2% from 2015 to 2016 in Rio de Janeiro^10^.

To effectively reduce new HIV infections among more vulnerable populations, the Joint United Nations Programme on HIV/AIDS (UNAIDS) recommends the promotion of combined prevention programs by integrating biomedical, behavioral, and structural intervention measures^11^. In this context, condoms are considered one of the most effective methods in preventing both HIV transmission and other sexually transmitted infections (STIs).

It is estimated that condoms were responsible for reversing 117 million new HIV infections worldwide from 1990 to 2020^12^. However, the prevalence of inconsistent condom use has been high among young and adult MSM^13^^), (^^14^. A study comparing sexual behaviors between two surveillance studies with MSM, using similar methods (that is, respondent-driven sampling [RDS]), estimated similar prevalences of unprotected receptive anal sex from 2009 to 2016: 35.2% and 36.4%, respectively. Nonetheless, when observing younger MSM aged 18 to 25 years, there is an increase in this prevalence: from 33.6% in 2009 to 41.8% in 2016^15^.

With TGW, the use of condoms in Brazil presents different proportions among the studies of the other regions investigated. A survey with *travestis* in the metropolitan area of Recife estimated that 87.3% of them had unprotected sex during their first sexual intercourse^16^. Moreover, in Salvador, two studies conducted with TGW found a prevalence of CAS of 73.4% in the last six months, from 2014 to 2016, and 74.8% in the previous thirty days, from 2016 to 2017^9^.

CAS is a practice that increases the risk of HIV infection when pre-exposure prophylaxis (PrEP) is not used, as well as other STIs^17^. Thus, understanding the explanatory factors of this behavior is important to develop intervention strategies for promoting sexual health and preventing HIV and other STIs. In 2009, Rocha et al.^13^, for instance, in a study with MSM, showed that illicit drug use, stable or commercial partnership, and friends that did not encourage condom use were factors associated with unprotected receptive anal sex. In addition, another study of young adult MSM in 2016 showed that unprotected receptive anal sex was associated with commercial sex, moderate or high perceived risk of HIV infection, homosexual identity, and poor self-rated health status, whereas, for older adult MSM, this practice was associated with a history of sexual violence, sex with younger partners, having had more than six sexual partners, and unprotected first sexual intercourse. In the same study, for both groups, being married or having a stable union were also factors associated with unprotected receptive anal sex^14^.

Among the TGW, there are recurrent processes of stigmatization, discrimination, and violence in the Brazilian context, which can hinder the negotiation of condom use^18^. Moreover, trust in the partner, depressive symptoms, and excessive use of psychoactive substance in specific contexts can also make it challenging to decide on condom use^19^.

In Brazil, research on the factors associated with CAS among adolescent MSM (AMSM) and adolescent TGW (ATGW) is limited to adolescents in the general population. Thus, this study seeks to analyze the factors that increase the practice of CAS among AMSM and ATGW in three Brazilian state capitals.

## METHODS

### Study design and population

This cross-sectional study, which used baseline data from the PrEP1519 cohort, was conducted from February 2019 to November 2021 and adopted convenience sampling of AMSM and ATGW aged 15 to 19. PrEP1519 is the first cohort study demonstrating the effectiveness of daily oral PrEP among MSM and TGW in Latin America, conducted in three Brazilian capitals: Belo Horizonte, Salvador, and São Paulo. The study population was composed of adolescents, self-identified as MSM or TGW, who reported living, working, or frequenting social spaces in one of these cities and who reported sexual relations that increase the risk of HIV infection with transgender women, *travestis*, or cisgender men at some point in their lives^20^. Adolescents with mental health issues or who were under the influence of drugs that compromised their decision to participate or their ability to respond to the interview or to receive clinical care were excluded. Other details are described in the study by Dourado et al.^20^.

### Data collection: recruitment and registration

Communication and demand strategies for PrEP were initiated in 2018 by mapping adolescent meeting places, such as schools and living spaces, as well as virtual platforms and partner search apps, such as Instagram, Facebook, WhatsApp, Grindr, and Badoo^20^. These strategies included information on sexual orientation and gender identity, sexual behavior, and HIV prevention, as well as the distribution of HIV self-testing, condoms, lubricants, and douching. In the case of online approaches, primarily implemented after the COVID-19 pandemic, prevention supplies were sent to participants by mail or distributed at PrEP services, according to participants’ preference and clinical assessment^21^. From mid-March 2020, in-person activities were adapted to online activities due to the COVID-19 pandemic^21^^)^ and resumed following local health protocols.

Participants could be enrolled in one of two groups of the study: PrEP and non-PrEP, which included the option of receiving other exclusive HIV prevention methods (i.e., testing, counseling, lubricant, HIV self-testing, etc.). Those who chose to use PrEP were evaluated for clinical eligibility criteria by a physician or nurse and returned after 30 days and every 90 days after that for follow-up at PrEP services. Those who did not opt for PrEP were advised to adhere to other methods of combined prevention. Testing for HIV, hepatitis A, B, and C, syphilis, and other bacterial STIs was provided to all participants in the initial and subsequent visits^20^.

### Instruments

This study was conducted using a socio-behavioral survey applied by healthcare professionals, peer educators, or trained researchers at the time of the participant’s enrollment in the cohort baseline. Data were recorded on an electronic virtual platform.

### Variables

The outcome variable was “condomless anal sex” (CAS). The question in the questionnaire refers to the practice of anal sex, insertive or receptive, without condom in the six months before the research, with casual or steady partners, and could be answered with yes or no.

The exposure variables were selected from the literature review and classified as follows:

- Sociodemographic: study population (AMSM; ATGW); age (18 and 19 years; 15 to 17 years); ethnicity/skin color (White, Black, Yellow, *Pardo* [Mixed-race] Indigenous); schooling (secondary or higher education in progress, complete elementary school.
- Behavioral: age at first sexual intercourse (older than 14 years, 14 years or younger); use of a condom during the first sexual intercourse (yes, no); age of the steady partner (more than five years, up to five years) use of psychoactive substances, classified as use of cocaine, marijuana, or ketamine (no, yes); history of group sex (no, yes); transactional sex defined as receiving gifts or money in exchange for sex (no, yes).
- Healthcare: type of medical follow-up (private, public); usual source of care (health post or center, hospital, physician, other).
- History of violence and discrimination based on gender or sexual orientation: (no, yes).

### Data analysis

A descriptive analysis was performed to estimate the prevalence of exposure and outcome variables. Additionally, the association between exposure variables and CAS was estimated using Pearson’s chi-square test and, when necessary, Fisher’s exact test, considering a statistical significance level of 5%. The variables that obtained a *p-*value<0.10 in this bivariate analysis were selected for multivariate analysis, which used a logistic regression model to estimate odds ratio for the association between the exposure variables and CAS. Subsequently, based on this logistic model, adjusted prevalence ratio (aPR) and 95% confidence intervals (95%CI) were estimated using the delta method, employing the *adjrr* command in the Stata statistical software version 14.0^22^^), (^^23^.

To choose the final model, the strategy of excluding variables was applied, initiating, one by one, the removal of less significant variables, i.e., with a *p-*value > 0.05. The exclusion of variables occurred in the following sequence: (i) having sexual intercourse at or before the age of 14; (ii) having used public services for care; and (iii) having experienced violence and discrimination based on gender identity and sexual orientation. The significance level of the *p-*value was 5%. The age variable was not statistically significant but was maintained in the final model due to its theoretical relevance. The model’s goodness of fit was analyzed using the Hosmer-Lemeshow test (*p-*value=0.64) and by examining the area under the ROC curve (AUC=0.65).

### Ethical aspects

For this study, the formative research protocol was approved by the Research Ethics Committees (RECs) of the World Health Organization (WHO) (protocol identification: “FIOTEC-PrEP Adolescent study”), the Faculty of Medicine of the University of São Paulo (USP) (no. 70798017.3.0000.0065), the Institute of Collective Health of the Federal University of Bahia (UFBA) (no. 01691718.1.0000.5030), and the Federal University of Minas Gerais (UFMG) (no. 17750313.0.0000.5149). Participants were informed about the research objectives and their rights when participating in the study. Those aged 18 years or older signed an informed consent form, whereas those aged from 15 to 17 years signed an informed assent form. In each city, there was a specific court decision regarding signing the informed consent form by the adolescent’s parents or guardians. In São Paulo, there was a judicial authorization to waive the informed consent form; in Salvador, waiver occurred in situations in which there was loss of family ties or in cases of risk of violence for the adolescent; in Belo Horizonte, exemption was not granted and written authorization from parents or guardians was required^24^.

## RESULTS

A total of 1,418 adolescents were included in the study: most were AMSM (91.5%), aged 18 to 19 years (75.9%), Black (40.5%), and with secondary or higher education in progress (92.7%). More than one-third of the participants reported having their first sexual intercourse at age 14 or younger (43.4%), and more than half said they did not use a condom during that intercourse (54.2%). Most participants reported having a relationship with steady partners younger than or equal to their age (82.1%), and almost half reported using psychoactive substances in the last three months (47.2%). About a quarter reported having participated in group sex (24.6%), 14.6% said they had engaged in transactional sex, and 32.2% said they had suffered discrimination and violence based on gender identity and sexual orientation. Moreover, 83.5% reported using the public sector for medical follow-up, and 43.6% used the health center as the usual source of care when sick (Table 1).

**Table 1:** Descriptive analysis of AMSM and ATGW characteristics and bivariate analysis of factors associated with condomless anal sex in the PrEP1519 study, Brazil, February 2019 to November 2021 (n=1,418).

Characteristic	n	%	Condomless anal sex		PR	95%CI	p-value
			Yes (n=1,143; 80.6%)				
			n	%			
**Sociodemographic**							
Study population							
Men who has sex with men	1,310	91.5	1,047	80.7	1.00		
*Travesti*/transgender women	121	8.5	96	79.3	0.98	0.89-1.08	0.718
Age							
18 to 19 years	1,084	75.9	861	80.0	1.00		
15 to 17 years	345	24.1	281	82.7	1.03	0.98-1.09	0.271
Ethnicity/skin color							
White	391	27.3	303	78.7	1.00		
Black	580	40.5	452	78.5	0.96	0.91-1.01	0.096
Yellow	26	1.8	19	73.1	0.91	0.72-1.14	0.381
*Pardo* (Mixed-race)	414	28.9	354	86.1	1.10	1.05-1.16	0.001
Indigenous	20	1.4	15	75.0	0.93	0.72-2.20	0.559
Schooling level							
Secondary or higher education in progress	1,191	92.7	952	80.0	1.00		
Complete elementary education	94	7.3	76	80.9	1.01	0.91-1.12	0.840
**Sexual behaviour**							
Age at first sexual intercourse							
>14 years	709	56.6	550	77.6	1.00		
≤14 years	543	43.4	455	84.0	1.08	1.03-1.14	0.004
Use of condom during the first sexual intercourse							
Yes	562	45.8	413	73.5	1.00		
No	665	54.2	575	86.5	1.18	1.11-1.24	0.001
Steady partner age							
More than 5 years	723	17.9	605	83.7	1.00		
Up to 5 years	158	82.1	128	81.0	1.03	0.95-1.12	0.434
Use of psychoactive substances in the last three months							
No	678	52.8	516	76.1	1.00		
Yes	605	47.2	510	84.4	1.11	1.05-1.17	≤0.001
Group Sex							
No	672	75.4	546	81.3	1.00		
Yes	219	24.6	197	90.0	1.11	1.04-1.17	≤0.001
Transactional sex							
No	1,049	85.4	823	78.5	1.00		
Yes	179	14.6	160	89.4	1.14	1.07-1.21	≤0.001
**Healthcare**							
Type of medical follow-up							
Private	236	16.5	176	75.2	1.00		
Public	1,195	83.5	967	81.7	1.09	1.00-1.17	0.033
Usual source of care							
Health post or center	561	43.6	454	81.2	1.00		
Hospital	416	32.4	327	78.6	0.97	0.92-1.03	0.375
Physician	82	6.4	60	73.2	0.91	0.79-1.04	0.142
Other	226	17.6	187	82.7	1.04	0.97-1.11	0.245
**Violence and discrimination based on gender identity or sexual orientation**							
Discrimination and violence							
No	832	67.8	652	78.4	1.00		
Yes	396	32.2	331	83.6	1.07	1.01-1.13	0.026

The prevalence of CAS in the last six months was 80.6% (95%CI 78.5%-82.6%). In the bivariate analysis, the factors that were positively associated with the outcome of the study were: self-reported *Pardo* (Mixed-race - PR: 1.10; 95%CI 1.05-1.16); having started sexual life at the age of 14 years or less (PR: 1.08; 95%CI 1.03-1.14); not having used a condom during the first sexual intercourse (PR: 1.18; 95%CI 1.11-1.24), use of psychoactive substances (PR: 1.11; 95%CI 1.05-1.17); having engaged in transactional sex (PR: 1.14; 95%CI 1.07-1.20); having used the public sector as usual source of care (PR: 1.09; 95%CI 1.07-1.21); and having experienced discrimination and violence based on gender identity or sexual orientation (PR: 1.07; 95%CI 1.01-1.13) (Table 1).

In the multivariate analysis, the factors that remained associated with CAS after adjusting the model were: condomless in the first sexual intercourse (aPR: 1.18; 95%CI 1.10-1.25); use of psychoactive substances in the last three months (aPR: 1.09; 95%CI 1.03-1.16); and having engaged in transactional sex (aPR: 1.11; CI95% 1.04-1.20). We also found that those aged 15 to 17 years had a higher prevalence of CAS compared with those aged 18 to 19 years (aPR: 1.07; 95%CI 0.99-1.13), although, in this case, there was no statistical significance at *p-*value 5% (Table 2).

**Table 2:** Multivariate analysis of factors associated with condomless anal sex among AMSM and ATGW in the PrEP1519 study, Brazil, February 2019 to November 2021.

Characteristic	Condomless anal sex		p-value
	aPR	95%CI	
Participant’s age^a^			
18 to 19 years	1.00		
15 to 17 years	1.07	0.99-1.13	0.051
Use of condoms during the first sexual intercourse			
Yes	1.00		
No	1.18	1.10-1.25	<0.001
Use of psychoactive substances in the last three months			
No	1.00		
Yes	1.09	1.03-1.16	0.003
Transactional sex			
No	1.00		
Yes	1.11	1.04-1.20	0.040

a Kept in the model due to its theoretical importance.

## DISCUSSION

This study estimated a higher prevalence of CAS among AMSM and ATGW compared to adolescents in Brazil’s general population (66.2%)^25^. Moreover, this prevalence was higher than that estimated in other studies among young and adult MSM and ATGW, such as the studies by Hentges et al.^26^^)^ on inconsistent use of condoms with casual partners among MSM in Brazil, by Magno et al.^18^^)^ on unprotected receptive anal sex among adult transgender women in Salvador, and by Satcher et al.^27^^)^ on unprotected insertive sex among transgender women in Peru. The high prevalence of CAS found in this study may indicate an inadequacy of condoms as a preventive method for this population, either due to decreasing information about the technique or due to the difficulty of consistent use caused by the sexual needs of this new generation.

CAS associated with the use of psychoactive substance and transactional sex may suggest a generational change related to the increase in sexual practices with a higher risk of STIs, as well as to the social vulnerability faced by this population. In this sense, it is necessary to consider other preventive methods, especially providing PrEP for HIV prevention, promoting testing and treatment of other STIs, and considering aspects of social vulnerability that hinder this population’s access to health supplies and services.

In this analysis, the age variable was not statistically significant. However, it was maintained in the final multivariate model due to its theoretical importance. For example, the studies by Queiroz et al.^28^^)^ and Rocha et al.^14^^)^ showed that younger adult MSM were more likely to report CAS than older adults. This can be explained by several factors, including issues related to stigma and discrimination, economic dependence that hinders access to condoms, and limitations of sexual health programs aimed at this specific group^29^.

This study also showed that the prevalence of CAS was higher among those who reported condomless sex during their first sexual intercourse compared with those who did the opposite. Condoms have been one of the most important technologies in combination with HIV prevention since the beginning of the AIDS pandemic and other STIs^11^. Although young people know the importance of its use, correct and consistent use is still a challenge^30^. Studies have shown that the onset of sexual life without a condom and the earlier onset of sexual life may be associated with decreased condom use during adulthood^31^.

Regarding psychoactive substances, this study showed a high prevalence of CAS among participants who reported consumption compared to those who did not. This finding corroborates the studies by Kapadia et al.^32^^)^ and Sousa et al.^33^ A systematic review with meta-analysis on the prevalence of psychoactive substance use among MSM in East and Southeast Asian countries also showed an association between drug use and condomless sex and HIV infection. The consumption of these substances may be motivated by recreation, intensification of sexual pleasure, and coping with social vulnerabilities^34^.

A higher prevalence of CAS was also found among the AMSM and ATGW, who reported transactional sex. Other studies conducted outside Brazil, such as those by Mgbako et al.^35^, Bórquez et al.^36^, and Beattie et al.^37^, showed similar findings. According to the 2021 UNAIDS report on HIV and sex work, transactional sex is consensual sex between two adults involving the exchange of money or other favors^38^. In the Brazilian context, transactional sex is not appropriate for adolescents under 18 years of age, constituting a crime due to sexual exploitation and representing a significant violation of human rights^39^. Baral et al.^40^, for example, showed that it is typical for the practice of transactional sex among adult MSM to begin when these men are still very young and is usually performed in contexts of sexual exploitation and violence. These contexts of vulnerability can lead to the worsening of depressive and anxiety symptoms, which, in turn, can interfere with the ability to negotiate the use of condoms in transactional sexual relations^41^, increasing the vulnerability of this population to HIV infection^38^.

Importantly, this research was conducted with AMSM and ATGW patients who were at high risk for HIV infection and other STIs and who sought a health service for the use of oral PrEP. Thus, the estimated high prevalence of CAS in the last six months may suggest that other prevention measures are preferred instead of condoms. On the other hand, it may also indicate the difficulty in accessing condoms faced by these young people, as well as the absence of sexual health education programs that promote the discussion of condom use among them. Therefore, it is recommended to strengthen sexual health education programs for young people that address the issue of sexuality and STI prevention, as well as to expand access to preventive methods, such as condoms and PrEP.

The main limitation of this study is that it was conducted with a hard-to-reach population using the convenience sampling technique, which, to a certain extent, may affect the sample size and analytical and statistical power of the sample in the three cities where the research was performed. Despite this, the study holds the advantage of covering adolescents at higher risk of HIV infection and who attend places of sociability, thus allowing a better understanding of the population groups that should be prioritized in public policies on HIV/AIDS.
