# Errata

**DOI:** 10.11606/s1518-8787.2025059005462err

**Published:** 2025-06-27

No artigo “Fatores associados ao sexo anal sem preservativo entre adolescentes homens que fazem sexo com homens e mulheres trans em três capitais brasileiras: estudo PrEP1519”, DOI http://doi.org/10.11606/s1518-8787.2020054005462, publicado na Revista de Saúde Pública. 2024;58: Suppl 1:8s, RSP corrige:

**Como citar (página 1):**

Onde se lê:

http://doi.org/10.11606/s1518-8787.2020054005462

Leia-se:

http://doi.org/10.11606/s1518-8787.2024058005462

**Rodapé (páginas 1 a 11):**

http://doi.org/10.11606/s1518-8787.2020054005462

Leia-se:

http://doi.org/10.11606/s1518-8787.2024058005462

