# Supplementary material for: Factors associated with condomless anal sex among adolescent men who have sex with men and transgender women in three Brazilian state capitals: a PrEP1519 study
Source: Rev Saude Publica. 2024 Oct 11;58(Suppl 1):8s. doi: 10.11606/s1518-8787.2024058005462 (PMC11573376; doi:10.11606/s1518-8787.2024058005462)
Supplement: Translation

# Translation: Fatores associados ao sexo anal sem preservativo entre adolescentes homens que fazem sexo com homens e mulheres trans em três capitais brasileiras: estudo PrEP1519

**Authors:** Rijone Rosário, Inês Dourado, Marcos Pereira, Lorenza Dezanet, Dirceu Greco, Alexandre Grangeiro, Laio Magno

**Affiliations:** I Universidade Federal da Bahia. Instituto de Saúde Coletiva. Salvador, BA, Brasil Universidade Federal da Bahia Instituto de Saúde Coletiva Salvador BA Brasil; II Fundação Oswaldo Cruz. Instituto René Rachou. Belo Horizonte, MG, Brasil Fundação Oswaldo Cruz Instituto René Rachou Belo Horizonte MG Brasil; III Universidade Federal de Minas Gerais. Faculdade de Medicina. Belo Horizonte, MG, Brasil Universidade Federal de Minas Gerais Faculdade de Medicina Belo Horizonte MG Brasil; IV Universidade de São Paulo. Faculdade de Medicina. São Paulo, SP, Brasil Universidade de São Paulo Faculdade de Medicina São Paulo SP Brasil; V Universidade do Estado da Bahia. Departamento de Ciências da Vida. Salvador, BA, Brasil Universidade do Estado da Bahia Departamento de Ciências da Vida Salvador BA Brasil

**Keywords:** Homens que fazem Sexo com Homens, Mulheres Trans, Adolescente, Sexo Anal sem Preservativo, HIV/Aids, Infecções Sexualmente Transmissíveis

## Abstract

**OBJETIVO:**

Analisar os fatores que aumentam a prática de sexo anal sem preservativo (SASP) entre adolescentes homens que fazem sexo com homens (aHSH) e travestis mulheres trans (aTrMT) em três capitais brasileiras.

**MÉTODOS:**

PrEP1519 é uma coorte prospectiva, multicêntrica, de demonstração da efetividade da profilaxia pré-exposição (PrEP) ao HIV entre aHSH e aTrMT, com idade entre 15 e 19 anos, em três capitais brasileiras. As análises foram realizadas com dados da linha de base da coorte, com a inclusão de 1.418 adolescentes inscritos de 2019 a 2021. O desfecho estudado foi SASP nos últimos seis meses, e os potenciais fatores associados foram sociodemográficos, comportamentais, de assistência à saúde e histórico de violência e discriminação. Foi realizada análise descritiva, bivariada e multivariada. Estimaram-se razões de prevalência ajustadas (RPa) e intervalos de confiança de 95% (IC95%).

**RESULTADOS:**

A maioria dos participantes era aHSH (91,5%), com idade de 18 a 19 anos (75,9%), pretos (40,5%), ensino médio e superior em andamento (92,7%), com relato de SASP na primeira relação sexual (54,2%), início da vida sexual antes dos 14 anos (43,4%) e história de sexo em grupo (24,6%) e de sexo transacional (14,6%). A prevalência de SASP nos últimos seis meses foi de 80,6% (IC95% 78,5%-82,6%). Adolescentes que relataram primeira relação sexual sem preservativo (RPa: 1,18; IC95% 1,10-1,25), consumo de substâncias psicoativas (RPa: 1,09; IC95% 1,03-1,16) e sexo transacional (RPa: 1,11; IC95% 1,04-1,20) tiveram maior prevalência de SASP nos últimos seis meses. Verificou-se também que aqueles com idade de 15 a 17 anos tiveram maior prevalência de SASP em comparação àqueles de 18 a 19 anos (RPa: 1,07; IC95% 0,99-1,13).

**CONCLUSÕES:**

A prevalência de SASP foi alta entre aHSH e aTrMT, bem como esteve associada a práticas que podem aumentar o risco de infecções sexualmente transmissíveis (IST). Assim, recomenda-se o fortalecimento de programas de saúde sexual para jovens que abordem o tema da sexualidade e prevenção de IST, tal como a ampliação do acesso a métodos preventivos, como preservativo e PrEP.

## INTRODUÇÃO

A adolescência é uma fase da vida caracterizada por mudanças físicas, psicológicas, cognitivas e de ampliação das interações sociais, na qual os adolescentes se veem diante de cobranças, desafios e novas experiências^1^. Nesse sentido, a educação em saúde sexual é recomendada para a promoção de informações que melhorem a qualidade de vida^2^.

No entanto, a educação em saúde sexual para adolescentes e jovens continua sendo um desafio tanto para as famílias quanto para os sistemas de educação e saúde, especialmente em países de baixa e média renda^3^. O Brasil, por exemplo, tem registrado diversos obstáculos na condução de programas de educação sexual nas escolas nos últimos anos, principalmente por conta do conservadorismo social^4^. Um estudo realizado em Salvador, Bahia, entre 2017 e 2018, evidenciou fragilidades no processo de desenvolvimento e integração de atividades de educação em saúde sexual em escolas públicas, principalmente pela dificuldade do diálogo entre os setores da saúde (i.e., Estratégia de Saúde da Família) e educação (i.e., escolas públicas) no âmbito do Programa Saúde na Escola (PSE). Além disso, esse estudo registrou experiências de racismo e discriminação relacionada à homossexualidade vivenciadas por adolescentes no ambiente escolar^3^.

Nesse contexto, o Brasil tem registrado aumento da taxa de incidência do vírus da imunodeficiência humana (HIV) entre adolescentes e jovens^5^. Também há uma preocupação especial por parte do Ministério da Saúde brasileiro com homens que fazem sexo com homens (HSH), travestis e mulheres trans (TrMT), visto que estudos de vigilância epidemiológica têm estimado alta prevalência do HIV nessas populações. Por exemplo, dois inquéritos nacionais realizados entre HSH adultos registraram aumento na prevalência de HIV, de 14,2% em 2009 para 18,4% em 2016^6^^), (^^7^. Entre TrMT adultas, a prevalência de HIV é desproporcionalmente maior do que aquela registrada entre a população em geral (0,6%) no período de 2001 a 2021^8^, com diferenças regionais e temporais importantes: 9% entre os anos de 2014 a 2016, e 24,3% entre os anos de 2016 e 2017, em Salvador^9^; e 31,2% (2015-2016) no Rio de Janeiro^10^.

Visando reduzir novas infecções por HIV de forma efetiva entre populações em situação de maior vulnerabilidade, o Programa Conjunto das Nações Unidas sobre HIV/AIDS (Unaids) preconiza a promoção de programas de prevenção combinada por meio da integração entre medidas de intervenção biomédicas, comportamentais e estruturais^11^. Nesse escopo, o preservativo é considerado um dos métodos mais eficazes na prevenção tanto da transmissão do HIV quanto de outras infecções sexualmente transmissíveis (IST).

Estima-se que o preservativo tenha sido responsável por reverter 117 milhões de novas infecções por HIV no mundo entre 1990 e 2020^12^. Entretanto, a prevalência do uso inconsistente de preservativo tem sido alta entre HSH jovens e adultos^13^^), (^^14^. Um estudo de comparação de comportamentos sexuais entre dois estudos de vigilância com HSH, utilizando métodos semelhantes (i.e., *respondent-driven sampling* - RDS), estimou prevalências similares de sexo anal receptivo sem preservativo entre 2009 e 2016: 35,2% e 36,4%, respectivamente. No entanto, ao observar HSH mais jovens, com idade de 18 a 25 anos, verifica-se crescimento dessa prevalência: de 33,6% em 2009 para 41,8% em 2016^15^

Com as TrMT, o uso de preservativo no Brasil apresenta proporções diferentes entre os estudos das diversas regiões investigadas. Estudo com travestis na região metropolitana de Recife estimou 87,3% de sexo sem uso de preservativo na primeira relação sexual^16^. Ademais, duas pesquisas com TrMT realizadas em Salvador estimaram prevalência de sexo anal sem preservativo (SASP) de 73,4% nos últimos seis meses entre 2014-2016, e 74,8% nos últimos trinta dias entre 2016 e 2017^9^.

SASP é uma prática que aumenta o risco de infecção por HIV na ausência de uso de profilaxia pré-exposição (PrEP), bem como de outras IST^17^. Desse modo, a compreensão dos fatores explicativos desse comportamento é importante para desenvolver estratégias de intervenção para a promoção da saúde sexual e prevenção de HIV e outras IST. Rocha et al.^13^, por exemplo, em estudo com HSH adultos em 2009, mostraram que o uso de drogas ilícitas, parceria estável ou comercial e comportamento de não incentivo ao uso de preservativo entre amigos eram fatores associados ao sexo anal receptivo sem preservativo. Além disso, outro estudo com HSH adultos jovens, em 2016, mostrou que sexo anal receptivo sem preservativo foi associado a sexo comercial, percepção de risco de infeção pelo HIV moderada ou alta, identidade homossexual e autoavaliação do estado de saúde ruim; enquanto, para os HSH adultos mais velhos, essa prática foi associada a história de violência sexual, sexo com parceiros mais jovens, ter tido mais de seis parceiros sexuais e primeira relação sexual sem preservativo. Nesse mesmo estudo, para ambos os grupos, ser casado ou ter uma união estável também foram fatores associados a sexo anal receptivo sem preservativo^14^.

Entre as TrMT, observam-se recorrentes processos de estigmatização, discriminação e violência no contexto brasileiro, os quais podem dificultar a negociação do uso de preservativo^18^. Além disso, confiança no parceiro, sintomas depressivos e consumo excessivo de álcool e drogas ilícitas em determinados contextos também podem dificultar a decisão sobre o uso de preservativo^19^.

No Brasil, pesquisas sobre os fatores associados a SASP entre aHSH e aTrMT são limitadas e se restringem a adolescentes da população geral. Desse modo, este artigo busca analisar os fatores que aumentam a prática de SASP entre aHSH e aTrMT em três capitais brasileiras.

## METODOLOGIA

### Desenho e população do estudo

Estudo transversal com dados da linha de base da coorte PrEP1519, realizado entre fevereiro de 2019 e novembro de 2021, que adotou amostragem de conveniência de aHSH e aTrMT, com idade entre 15 e 19 anos. O PrEP1519 é o primeiro estudo de coorte de demonstração da efetividade da PrEP oral de uso diário entre aHSH e aTrMT na América Latina, sendo conduzido em três capitais brasileiras: Belo Horizonte, Salvador e São Paulo. A população do estudo foi composta por adolescentes, autoidentificados como HSH ou TrMT, que declararam morar, trabalhar ou frequentar espaços de sociabilidade em alguma dessas cidades e que reportaram relações sexuais que aumentam o risco de infeção por HIV com mulheres trans, travestis ou homens cisgêneros alguma vez na vida^20^. Foram excluídos adolescentes com questões de saúde mental e/ou que se apresentaram sob efeito de drogas que comprometessem a decisão de participação, a realização de entrevista ou o atendimento clínico. Outros detalhes estão descritos no estudo de Dourado et al.^20^.

### Coleta de dados: recrutamento e inscrição

As estratégias de comunicação e criação de demanda para a PrEP foram iniciadas em 2018 por meio do mapeamento de locais de encontro dos adolescentes, como escolas e espaços de convivência, bem como plataformas virtuais e aplicativos de busca de parceiros como Instagram, Facebook, WhatsApp, Grindr e Badoo^20^. As estratégias de criação de demanda incluíram informações sobre orientação sexual e identidade de gênero, comportamento sexual e prevenção do HIV, tal qual a distribuição de autoteste de HIV, preservativos, lubrificantes e ducha higiênica. No caso de abordagens *on-line*, especialmente implementadas após a pandemia de covid-19, os suprimentos de prevenção foram enviados aos participantes por correio ou retiradas nos serviços de PrEP, de acordo com a preferência dos participantes e avaliação clínica^21^. A partir de meados de março de 2020, as atividades presenciais foram adaptadas para atividades *on-line* devido à pandemia de covid-19^21^^)^ e, depois, retomadas de acordo com os protocolos sanitários locais.

Os participantes poderiam ser inscritos em um dos dois braços da pesquisa: o de PrEP e o de não PrEP, que incluiu a opção de recebimento exclusivo de outros métodos de prevenção de HIV (i.e., testagem, aconselhamento, lubrificante, autoteste de HIV etc.). Aqueles que escolhiam usar a PrEP passavam por avaliação dos critérios de elegibilidade clínica por médico ou enfermeiro e retornavam após trinta dias e, posteriormente, a cada noventa dias para o acompanhamento nos serviços de PrEP. Aqueles que não optavam pela PrEP eram aconselhados a aderir a outros métodos de prevenção combinada. Foram ofertadas testagens para HIV, hepatite A, B e C, sífilis e outras IST bacterianas para todos os participantes nas visitas iniciais e subsequentes^20^.

### Instrumentos

O instrumento utilizado neste estudo foi questionário sociocomportamental aplicado por profissional de saúde, educadores de pares ou pesquisadores treinados no momento da inscrição do participante na linha de base da coorte. Os dados foram registrados em plataforma virtual eletrônica.

### Variáveis

Para este estudo, a variável “sexo anal sem preservativo” foi usada como desfecho. A pergunta no questionário se refere à prática de sexo anal, insertivo ou receptivo, sem preservativo nos seis meses anteriores à pesquisa, com parceiros casuais ou fixos, e classificada em sim e não.

As variáveis de exposição foram selecionadas a partir da revisão de literatura e classificadas em:

- Sociodemográficas: população de estudo (aHSH, aTrMT); idade (18 e 19 anos, 15 a 17 anos); raça/cor de pele (branca, preta, amarela, parda, indígena); escolaridade (ensino médio e superior em andamento, ensino fundamental concluído).
- Comportamentais: idade na primeira relação sexual (maior que 14 anos, menor ou igual a 14 anos); uso de preservativo na primeira relação sexual (sim, não); idade do parceiro fixo (cinco anos mais velho ou mais que o participante, menor ou igual à do participante); consumo de substâncias psicoativas, classificado como uso de cocaína, maconha ou cetamina (não, sim); histórico de sexo em grupo (não, sim); sexo transacional definido por receber presentes ou dinheiro em troca de sexo (não, sim).
- Assistência de saúde: acompanhamento médico (privado, público); fonte usual de cuidado (posto ou centro de saúde, hospital, médico, outro).
- Histórico de violência e discriminação de gênero ou orientação sexual: (não, sim).

### Análise de dados

Foi realizada análise descritiva com estimativa das prevalências das variáveis de exposição e desfecho. Além disso, testou-se a associação entre as variáveis de exposição e SASP por meio do teste de qui-quadrado de Pearson e, quando necessário, do teste exato de Fisher, considerando o nível de significância estatística de 5%. As variáveis que obtiveram valor de *p<*0,10 nessa análise bivariada foram selecionadas para a análise multivariada, que utilizou um modelo de regressão logística para estimar *odds ratio* da associação entre as variáveis de exposição e SASP. Posteriormente, com base nesse modelo logístico, as razões de prevalência ajustadas (RPa) e intervalos de confiança a 95% (IC95%) foram estimados pelo método delta, utilizando o comando *adjrr* no pacote estatístico Stata versão 14.0^22^^), (^^23^.

Para a escolha do modelo final, foi aplicada a estratégia de exclusão de variáveis, iniciando, uma a uma, a remoção das variáveis menos significativas, ou seja, com valor de *p*>0,05. A exclusão das variáveis ocorreu na sequência: (i) ter vida sexual antes ou até os 14 anos; (ii) ter usado o serviço público para atendimento; e (iii) ter sofrido violência e discriminação baseada em identidade de gênero e orientação sexual. O nível de significância do valor de *p* foi de 5%. A variável idade não teve significância estatística, mas foi mantida no modelo final por apresentar relevância teórica. A bondade do ajuste do modelo foi analisada pelo teste de Hosmer-Lemeshow *(*valor de *p=*0,64*)* e por meio da área da curva ROC (AUC=0,65)*.*

### Aspectos éticos

Para este estudo, o protocolo da pesquisa formativa foi aprovado pelos Comitês de Ética em Pesquisa (CEP) da Organização Mundial de Saúde (OMS) (identificação do protocolo: “Fiotec-PrEP Adolescent study”), da Faculdade de Medicina da Universidade de São Paulo (USP) (nº 70798017.3.0000.0065), do Instituto de Saúde Coletiva da Universidade Federal da Bahia (UFBA) (nº 01691718.1.0000.5030) e da Universidade Federal de Minas Gerais (UFMG) (nº 17750313.0.0000.5149). Os participantes tiveram informação sobre os objetivos da pesquisa e seus direitos ao participar no estudo. Aqueles com idade de 18 anos ou mais assinaram termo de consentimento livre e esclarecido (TCLE), enquanto aqueles com idade entre 15 e 17 anos assinaram termo de assentimento. Em cada cidade houve uma decisão judicial específica sobre a assinatura do TCLE pelos pais ou responsáveis pelo adolescente. Em São Paulo, houve autorização judicial de dispensa do TCLE; em Salvador, a dispensa ocorreu em situações em que havia perda de vínculo familiar ou em casos de risco de violência para o adolescente; em Belo Horizonte, a dispensa não foi concedida e foi necessária a autorização por escrito dos pais ou responsáveis^24^.

## RESULTADOS

Foram incluídos 1.418 adolescentes no estudo: a maioria era aHSH (91,5%), com idade de 18 a 19 anos (75,9%), raça/cor de pele preta (40,5%) e com ensino médio e superior em andamento (92,7%). Mais de um terço dos participantes declarou ter tido a primeira relação sexual com idade menor ou igual a 14 anos (43,4%), e mais da metade disse não ter usado preservativo nessa relação (54,2%). A maioria dos participantes declarou se relacionar com parceiros fixos com idade menor ou igual a deles (82,1%), e quase a metade declarou consumo de substâncias psicoativas nos últimos três meses (47,2%). Cerca de um quarto declarou ter participado de sexo em grupo (24,6%), 14,6% disseram ter realizado sexo transacional, e 32,2% revelaram ter sofrido discriminação e violência baseada em identidade de gênero e orientação sexual. Além disso, 83,5% relataram usar o setor público para acompanhamento médico, e 43,6% usam o posto ou centro de saúde como fonte usual de cuidado quando doentes (Tabela 1).

**Tabela 1:** Análise descritiva das características dos aHSH e TrTM e análise bivariada de fatores associados a sexo anal sem preservativo no estudo PrEP1519, Brasil, fevereiro de 2019 a novembro de 2021 (n=1.418).

Variável	n	%	Sexo anal sem preservativo		RP	IC95%	Valor de p
			Sim (n=1.143; 80,6%)				
			n	%			
**Sociodemográficas**							
População de estudo							
Homem que fazem sexo com homem	1.310	91,5	1.047	80,7	1,00		
Travesti/mulher travesti	121	8,5	96	79,3	0,98	0,89-1,08	0,718
Idade							
18 anos a 19 anos	1.084	75,9	861	80,0	1,00		
15 anos a 17 anos	345	24,1	281	82,7	1,03	0,98-1,09	0,271
Raça/cor de pele							
Branca	391	27,3	303	78,7	1,00		
Preta	580	40,5	452	78,5	0,96	0,91-1,01	0,096
Amarela	26	1,8	19	73,1	0,91	0,72-1,14	0,381
Parda	414	28,9	354	86,1	1,10	1,05-1,16	0.001
Indígena	20	1,4	15	75,0	0,93	0,72-2,20	0,559
Escolaridade							
Médio e superior em andamento	1.191	92,7	952	80,0	1,00		
Fundamental concluído	94	7,3	76	80,9	1,01	0,91-1,12	0,840
**Comportamento sexual**							
Idade na primeira relação sexual							
>14 anos	709	56,6	550	77,6	1,00		
<=14 anos	543	43,4	455	84,0	1,08	1,03-1,14	0,004
Uso de preservativo na primeira relação sexual							
Sim	562	45,8	413	73,5	1,00		
Não	665	54,2	575	86,5	1,18	1,11-1,24	0.001
Idade do parceiro fixo							
Até cinco anos mais velho ou mais	723	17,9	605	83,7	1,00		
Menor ou igual a mesma idade	158	82,1	128	81,0	1,03	0,95-1,12	0,434
Consumo de substâncias psicoativas nos últimos três meses							
Não	678	52,8	516	76,1	1,00		
Sim	605	47,2	510	84,4	1,11	1,05-1,17	≤0,001
Sexo em grupo							
Não	672	75,4	546	81,3	1,00		
Sim	219	24,6	197	90,0	1,11	1,04-1,17	≤0,001
Sexo transacional							
Não	1.049	85,4	823	78,5	1,00		
Sim	179	14,6	160	89,4	1,14	1,07-1,21	≤0,001
**Assistência de saúde**							
Acompanhamento médico							
Privado	236	16,5	176	75,2	1,00		
Público	1.195	83,5	967	81,7	1,09	1,00-1,17	0,033
Fonte usual de cuidado							
Posto ou centro de saúde	561	43,6	454	81,2	1,00		
Hospital	416	32,4	327	78,6	0,97	0,92-1,03	0,375
Médico	82	6,4	60	73,2	0,91	0,79-1,04	0,142
Outro	226	17,6	187	82,7	1,04	0,97-1,11	0,245
**Violência e discriminação baseada na identidade de gênero ou orientação sexual**							
Discriminação e violência							
Não	832	67,8	652	78,4	1,00		
Sim	396	32,2	331	83,6	1,07	1,01-1,13	0,026

A prevalência de SASP nos últimos seis meses foi de 80,6% (IC95% 78,5%-82,6%). Na análise bivariada, os fatores que se associaram positivamente ao desfecho do estudo foram: autorrelato de cor de pele parda (RP: 1,10; IC95% 1,05-1,16); ter iniciado a vida sexual com idade menor ou igual a 14 anos (RP: 1,08; IC95% 1,03-1,14); não ter usado preservativo na primeira relação sexual (RP: 1,18; IC95% 1,11-1,24); consumo de substâncias psicoativas nos últimos três meses (RP: 1,11; IC95% 1,05-1,17); ter praticado sexo transacional (RP: 1,14; IC95% 1,07-1,20); ter utilizado o setor público como fonte usual de cuidado (RP: 1,09; IC95% 1,07-1,21); e ter sofrido discriminação e violência baseada na identidade de gênero ou orientação sexual (RP: 1,07; IC95% 1,01-1,13) (Tabela 1).

Na análise multivariada, os fatores que permaneceram associados a SASP após o ajuste do modelo foram: não ter usado preservativo na primeira relação sexual (RPa: 1,18; IC95% 1,10-1,25); consumo de substâncias psicoativas nos últimos três meses (RPa: 1,09; IC95% 1,03-1,16); e ter praticado sexo transacional (RPa: 1,11; IC95% 1,04-1,20). Adicionalmente, verificou-se que adolescentes com idade de 15 a 17 anos tiveram maior prevalência de SASP em comparação àqueles de 18 a 19 anos (RPa: 1,07; IC95% 0,99-1,13), embora, neste caso, não tenha tido significância estatística ao nível do *p* valor de 5% (Tabela 2).

**Tabela 2:** Análise multivariada dos fatores associados a sexo anal sem preservativo entre aHSH e aTrMT no estudo PrEP1519, Brasil, fevereiro de 2019 a novembro de 2021.

Variável	Sexo anal sem preservativo		Valor de p
	RPa	IC95%	
Idade do participante^a^			
18 a 19 anos	1,00		
15 a 17anos	1,07	0,99-1,13	0,051
Uso de preservativo na primeira relação sexual			
Sim	1,00		
Não	1,18	1,10-1,25	<0,001
Consumo de drogas ilícitas			
Não	1,00		
Sim	1,09	1,03-1,16	0,003
Sexo transacional			
Não	1,00		
Sim	1,11	1,04-1,20	0,040

a Mantida no modelo devido a sua importância teórica.

## DISCUSSÃO

Este estudo estimou alta prevalência de SASP entre aHSH e aTrTM quando comparado ao não uso de preservativo entre adolescentes da população geral no Brasil (66,2%)^25^. Ademais, essa prevalência foi maior do que aquela estimada em outras pesquisas entre HSH e TrMT jovens e adultos, como os estudos de Hentges et al.^26^^)^ sobre uso inconsistente de preservativo com parceiros casuais entre HSH no Brasil, de Magno et al.^18^^)^ sobre sexo anal receptivo sem preservativo entre mulheres trans adultas em Salvador e de Satcher et al.^27^^)^ acerca de sexo insertivo sem preservativo entre mulheres trans no Peru. A alta prevalência de SASP encontrada neste estudo pode indicar uma inadequação do preservativo como método preventivo para essa população, quer por uma redução da informação sobre o método ou pela dificuldade de uso consistente em relação às necessidades sexuais dessa nova geração.

SASP associado a consumo de substâncias psicoativas e a sexo transacional pode estar indicando uma mudança geracional relacionada ao aumento de práticas sexuais com maior risco de IST, bem como à vulnerabilidade social enfrentada por essa população. Nesse sentido, é necessário considerar outros métodos preventivos, especialmente a oferta de PrEP para prevenção do HIV, a ampliação da testagem e tratamento de outras IST e a consideração de aspectos de vulnerabilidade social que dificultam o acesso dessa população a insumos e serviços de saúde.

Nesta análise, a variável idade não teve significância estatística. Entretanto, foi mantida no modelo multivariado final em razão de sua importância teórica. Os estudos de Queiroz et al.^28^^)^ e Rocha et al.^14^, por exemplo, mostraram que HSH adultos mais jovens tinham maior chance de relatar SASP do que os mais velhos. Isso pode ser explicado por diversos fatores, que incluem questões relacionadas a estigma e discriminação, dependência econômica que dificulta o acesso a preservativos e limitações de programas de saúde sexual voltadas a esse grupo específico^29^.

Esta pesquisa também mostrou que a prevalência de SASP foi maior entre os que declararam não ter usado preservativo na primeira relação sexual, comparado àqueles que relataram ter usado. O preservativo é uma das tecnologias mais importantes na prevenção combinada do HIV desde o início da pandemia da síndrome da imunodeficiência adquirida (aids) e de outras IST^11^. Embora os jovens conheçam a importância de seu uso, o uso correto e consistente ainda é um desafio^30^. Estudos têm mostrado que o início da vida sexual sem preservativo e o início da vida sexual mais cedo podem estar associados à diminuição do uso de preservativo durante a vida adulta^31^.

No que diz respeito a substâncias psicoativas, este estudo mostrou uma prevalência alta de SASP entre participantes que declararam consumo em comparação àqueles que não teriam consumido. Esse achado corrobora os estudos de Kapadia et al.^32^^)^ e Sousa et al.^33^ Uma revisão sistemática com meta-análise sobre a prevalência do uso de substâncias psicoativas entre HSH em países do leste e sudeste asiático também mostrou associação entre o uso de drogas e a prática de sexo sem preservativo e com a infeção por HIV. O consumo dessas substâncias pode ser motivado por recreação, intensificação do prazer sexual e enfrentamento de vulnerabilidades sociais^34^.

Maior prevalência de SASP foi também encontrada entre os aHSH aTrMT que tiveram o relato de sexo transacional. Outras pesquisas realizadas fora do Brasil, como os estudos de Mgbako et al.^35^, Bórquez et al.^36^^)^ e Beattie et al.^37^, tiveram achados semelhantes. Segundo o relatório da Unaids sobre HIV e trabalho sexual de 2021, o sexo transacional é o sexo consensual entre duas pessoas adultas envolvendo a troca de dinheiro ou outros favores^38^. No contexto brasileiro, o termo sexo transacional não se adequa aos adolescentes menores de 18 anos, constituindo crime por se tratar de exploração sexual e representar grande violação dos direitos humanos^39^. Baral et al.^40^, por exemplo, mostraram que não é incomum a prática de sexo transacional entre HSH adultos começar quando esses homens ainda são muito jovens, sendo realizada geralmente em contextos de exploração sexual e violência. Esses contextos de vulnerabilidade podem ocasionar o agravamento de sintomas depressivos e de ansiedade que, por sua vez, podem interferir na capacidade de negociação do uso de preservativo nas relações sexuais transacionais^41^, aumentando a vulnerabilidade dessa população à infeção pelo HIV^38^.

É importante ressaltar que esta pesquisa foi realizada com aHSH aTrMT que estavam em alto contexto de risco para infecção pelo HIV e outras IST e que procuraram um serviço de saúde para o uso de PrEP oral. Desse modo, por um lado, a alta prevalência estimada de SASP nos últimos seis meses pode sugerir uma dificuldade do uso de preservativo como método de prevenção pela disponibilidade de outra medida de prevenção. Por outro lado, pode também indicar a dificuldade de acesso desses jovens aos preservativos, bem como a ausência de programas de saúde sexual que promovam a discussão do uso de preservativo entre esses jovens. Portanto, recomenda-se o fortalecimento de programas de saúde sexual para jovens que abordem o tema da sexualidade e prevenção de IST, assim como a ampliação do acesso a métodos preventivos, como preservativo e PrEP.

A principal limitação deste estudo é ter sido realizado em uma população de difícil acesso usando a técnica de amostragem por conveniência, o que, de certa forma, pode afetar o tamanho amostral e o poder estatístico analítico da população estudada nas três cidades nas quais decorreu a pesquisa. Apesar disso, o estudo teve uma vantagem de abranger adolescentes com maior risco de infecção de HIV e que frequentam locais de sociabilidade, permitindo, assim, uma melhor compreensão dos grupos populacionais que devem ser priorizados nas políticas públicas de HIV/aids.
