# Supplementary material for: Errata
Source: Rev Saude Publica. 2025 Jun 27;59:e8serr. [Article in Portuguese] doi: 10.11606/s1518-8787.2025059005462err (PMC12219643; doi:10.11606/s1518-8787.2025059005462err)
Supplement: Related texts

# Correction: Erratum

In the article “Factors associated with condomless anal sex among adolescent men who have sex with men and transgender women in three Brazilian state capitals: a PrEP1519 study”, DOI http://doi.org/10.11606/s1518-8787.2020054005462, published on the Revista de Saúde Pública. 2024;58: Suppl 1:8s, RSP corrects:

**How to cite (page 1):**

Where it reads:

http://doi.org/10.11606/s1518-8787.2020054005462

It should read:

http://doi.org/10.11606/s1518-8787.2024058005462

**Footnote (pages 1 to 11):**

Where it reads:

http://doi.org/10.11606/s1518-8787.2020054005462

It should read:

http://doi.org/10.11606/s1518-8787.2024058005462
